# Rapid Antigen Test Combined with Chest Computed Tomography to Rule Out COVID-19 in Patients Admitted to the Emergency Department

**DOI:** 10.3390/jcm10163455

**Published:** 2021-08-04

**Authors:** Sabrina Kepka, Mickaël Ohana, François Séverac, Joris Muller, Eric Bayle, Yvon Ruch, Elodie Laugel, Mathieu Oberlin, Morgane Solis, Yves Hansmann, Pascal Bilbault, Samira Fafi Kremer

**Affiliations:** 1 Emergency Department, Hôpitaux Universitaires de Strasbourg, 1 Place de l’Hôpital, 67091 Strasbourg, France; eric.bayle@chru-strasbourg.fr (E.B.); mathieu.oberlin@chru-strasbourg.fr (M.O.); pascal.bilbault@chru-strasbourg.fr (P.B.); 2 URCEco, Hôtel Dieu, AP-HP, 1 Place du Parvis Notre Dame, 75004 Paris, France; 3 Radiology Department, Nouvel Hôpital Civil, Hôpitaux Universitaires de Strasbourg, 1 Place de l’Hôpital, 67091 Strasbourg, France; mickael.ohana@chru-strasbourg.fr; 4 Groupe Méthodes en Recherche Clinique (GMRC), Hôpitaux Universitaires de Strasbourg, 1 Place de l’Hôpital, 67091 Strasbourg, France; francois.severac@chru-strasbourg.fr; 5 Public Health Department, Hôpitaux Universitaires de Strasbourg, 1 Place de l’Hôpital, 67091 Strasbourg, France; joris.muller@chru-strasbourg.fr; 6 Department of Infectious and Tropical Diseases, Hôpitaux Universitaires de Strasbourg, 1 Place de l’Hôpital, 67091 Strasbourg, France; yvon.ruch@chru-strasbourg.fr (Y.R.); yves.hansmann@chru-strasbourg.fr (Y.H.); 7 Fédération de Médecine Translationnelle de Strasbourg, Université de Strasbourg, 67000 Strasbourg, France; elodie.laugel@chru-strasbourg.fr (E.L.); morgane.solis@chru-strasbourg.fr (M.S.); samira.fafi-kremer@chru-strasbourg.fr (S.F.K.); 8 Department of Virology, Hôpitaux Universitaires de Strasbourg, 1 Place de l’Hôpital, 67091 Strasbourg, France; 9 Department of Internal Medicine, Hôpitaux Universitaires de Strasbourg, 1 Place de l’Hôpital, 67091 Strasbourg, France; 10 UMR 1260, INSERM/Université de Strasbourg CRBS, 1 rue Eugene Boeckel, 67000 Strasbourg, France

**Keywords:** SARS-CoV2, antigen-detecting rapid diagnostic test, RT-PCR, emergency department, chest computed tomography

## Abstract

Objective: Correct and timely identification of SARS-CoV-2-positive patients is critical in the emergency department (ED) prior to admission to medical wards. Antigen-detecting rapid diagnostic tests (Ag-RDTs) are a rapid alternative to Reverse-transcriptase polymerase chain reaction (RT-PCR) for the diagnosis of COVID-19 but have lower sensitivity. Methods: We evaluated the performance in real-life conditions of a strategy combining Ag-RDT and chest computed tomography (CT) to rule out COVID-19 infection in 1015 patients presenting in the ED between 16 November 2020 and 18 January 2021 in order to allow non-COVID-19 patients to be hospitalized in dedicated units directly. The combined strategy performed in the ED for patients with COVID-19 symptoms was assessed and compared with RT-PCR. Results: Compared with RT-PCR, the negative predictive value was 96.7% for Ag-RDT alone, 98.5% for Ag-RDT/CT combined, and increased to 100% for patients with low viral load. Conclusion: A strategy combining Ag-RDT and chest CT is effective in ruling out COVID-19 in ED patients with high precision.

## 1. Introduction

Correct and timely identification of SARS-CoV-2-positive patients is critical in the emergency department (ED) prior to admission to medical wards. Reverse-transcriptase polymerase chain reaction (RT-PCR) is the diagnostic gold standard, but is time-consuming and affects the length of stay in the ED. However, a prolonged length of stay in the ED is known to be associated with adverse events [1,2]. Furthermore, an inefficient strategy of triage during the pandemic may impact the quality of care for patients that consult the ED with no symptoms of COVID, such as chest pain [3]. Antigen-detecting rapid diagnostic tests (Ag-RDTs) are a simple and rapid alternative to nucleic acid amplification assays and can provide results within 15–20 min, thereby reducing the time needed to make clinical decisions in the ED. The reported lower sensitivity (56.2–68.6%, with RT-PCR as a reference) does not allow the use of Ag-RDT alone as frontline testing for diagnosing coronavirus disease 2019 (COVID 19) in the ED [4,5,6,7]. Chest computed tomography (CT) is an alternative rapid diagnostic method in the ED for triage, with high specificity for symptomatic patients, ranging from 83.5% to 96.2% (pooled specificity of 91.3% (95% CI 87.6–94.0) in a Cochrane meta-analysis) compared to RT-PCR [8,9,10]. CT has been reported to efficiently complement RT-PCR in diagnosing COVID-19, especially with access to ultra-low-dose CT [9].

At the Strasbourg University hospitals, Ag-RDTs are performed for all patients admitted to the ED with suspected COVID-19 or before transfer to medical wards. Patients with a positive Ag-RDT are transferred to the medical unit dedicated to COVID-19. Patients with a negative Ag-RDT and suspected COVID-19 undergo a chest CT and RT-PCR test before clinical decision-making, leading to slower patient flows. To investigate whether patients can be triaged in the ED using a combination of Ag-RDT and chest CT without RT-PCR, we evaluated the performance of Ag-RDT/chest CT with and without RT-PCR in real-life conditions.

## 2. Materials and Methods

We conducted a retrospective study in the ED of Strasbourg University hospitals between 16 November 2020 and 18 January 2021. Since 16 November 2020, Public Health France (the French national public health agency) has considered that a positive Ag-RDT can diagnose a patient as a confirmed case of COVID-19.

The study included patients older than 18 years of age and admitted to the ED with COVID-19-like symptoms and a negative Ag-RDT. There were no exclusion criteria.

The study outcome was the diagnostic performance in real-life conditions of a strategy combining Ag-RDT and chest CT to identify patients with COVID-19 presenting in the ED with or without RT-PCR to allow non-COVID-19 patients to be hospitalized in the dedicated unit directly.

We considered body temperatures > 38 °C, respiratory signs, other signs (diarrhea, confusion, etc.), an age older than 85 years, or living in an institution as suspected indicators of COVID-19.

The study considered RT-PCR the gold standard for diagnosing COVID-19. The combined Ag-RDT/CT strategy performed in the ED for patients with COVID-19 symptoms was assessed in comparison with RT-PCR.

To detect SARS-CoV-2 antigens on nasopharyngeal swabs, we employed the Panbio™ COVID-19 Ag Rapid Test Device (Abbott Diagnostic GmbH, Jena, Germany), an immunochromatographic test with a membrane strip precoated with antibodies to the SARS-CoV-2 nucleocapsid. The kit was used according to the manufacturer’s instructions. After collecting samples for the Ag-RDT, nasopharyngeal specimens were transported to the virology laboratory of the University Hospital of Strasbourg for RT-PCR analysis. The primer and probe sequences targeted 2 regions in the RdRp gene specific to SARS-CoV-2. Assay sensitivity is approximately 10 copies/reaction (Institut Pasteur, Paris, France) [11] The RT-PCR results were reported by cycle threshold (Ct). Ct levels are inversely proportional to the amount of target nucleic acid in the sample.

The CT scans were reviewed by an expert thoracic radiologist and classified into 3 categories following the European Society of Radiology and European Society of Thoracic Imaging guidelines [12]: *compatible*, for typical COVID-19 lesions; *incompatible*, when no infectious sign was present; and *indeterminate*. Typical chest CT findings included bilateral ground-glass opacities with peripheral distribution, interlobular septal thickening, and subpleural alveolar consolidations. Indeterminate findings included bronchiolitis, centrilobular nodules, and single lobar consolidation.

We evaluated the predictive negative value of the strategy combining Ag-RDT/CT for diagnosing COVID-19 compared with RT-PCR. The combined Ag-RDT/CT strategy was considered as negative for patients with negative Ag-RDT and CT incompatible or indeterminate. We extracted data from the electronic health records using the SQL language. We used Bayesian inference to estimate the probabilities and Jeffreys prior (a beta [0.5, 0.5] distribution) on the probability of interest to determine a two-sided 95% confidence interval from a beta posterior. All analyses were performed using R software version 4.0.3. (R Development Core Team 2020).

The study was conducted in accordance with the principles set forth by Good Clinical Practice guidelines and the Declaration of Helsinki. It was approved by the Ethics Committee of the University of Strasbourg (CE No.-2020-43). In accordance with French legislation, formal written informed consent was not required for this type of study because data were entirely retrospectively studied [13]. A non-opposition procedure was used and no patient expressed opposition to the use of his or her medical data for research purposes.

## 3. Results

**Figure 1:**
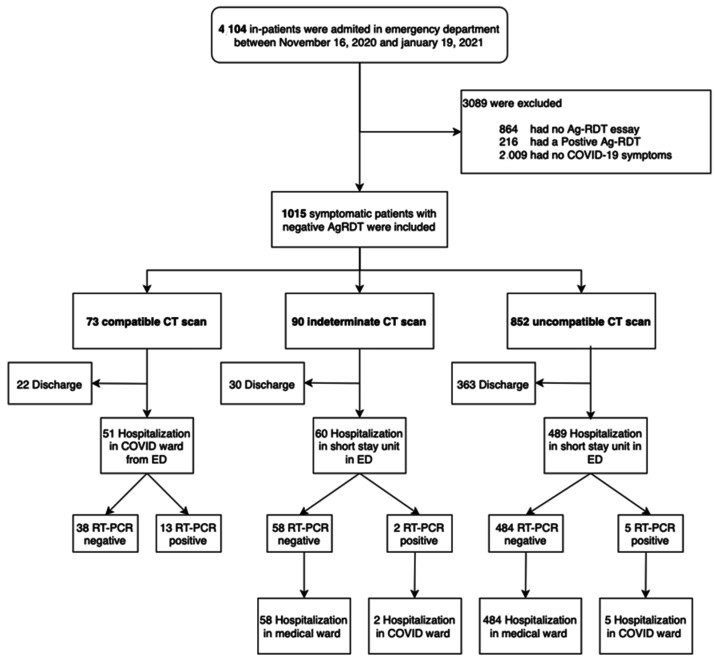
Management of patients with COVID symptoms in Emergency Departments. Ag-RDT: Antigen-detecting rapid diagnostic tests; CT: Computed Tomography; ED: Emergency Department; RT-PCR: Reverse-transcriptase polymerase chain reaction.

During the study period, 4104 patients were admitted to the ED (Figure 1); 3240 underwent Ag-RDTs, and 216 had positive Ag-RDTs.

**Figure 2:**
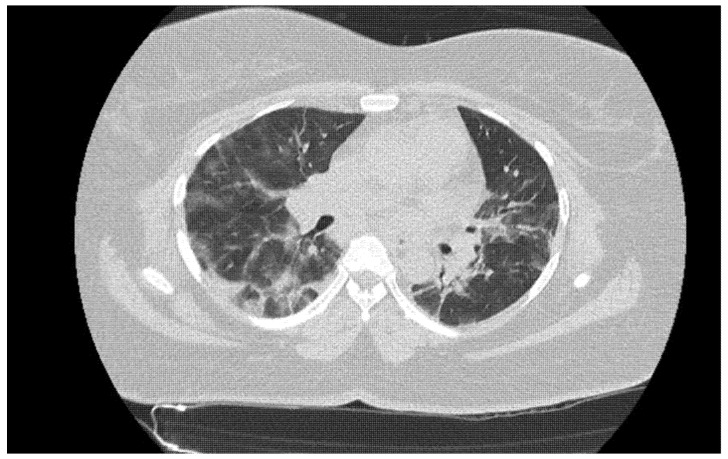
Unenhanced chest CT in a 44-year old-woman with dyspnea and fever for the previous 3 days. Bilateral ground-glass opacities are present, with subpleural predominance and associated interstitial septal thickening. Findings were consistent with typical COVID-19 pneumonia, encompassing 25 to 50% of the total lung parenchyma.

A total of 1015 patients (458 [45.1%] women, 557 [54.9%] men; median age, 70.4 years (SD 18.2)) with a negative Ag-RDT underwent a chest CT in the ED; 73 had a compatible CT (Figure 2), 90 had an indeterminate CT, and 852 had an incompatible CT (Table 1). The overall prevalence of SARS-CoV-2 infection was 10.06% in the ED during the study.

The time since onset of symptoms for patients was from 8 to 15 days for patients with positive RT-PCR (data available for 6 patients). One patient revealed a contact with a COVID patient 6 days earlier, and 17 had a positive RT-PCR from 6 to 18 days prior to their ED stay. When considering the CT results, the time since onset of symptoms was from 8 to 15 days in the group with compatible CT results, and from 8 to 9 days in the group with indeterminate or incompatible CT results.

**Figure 3:**
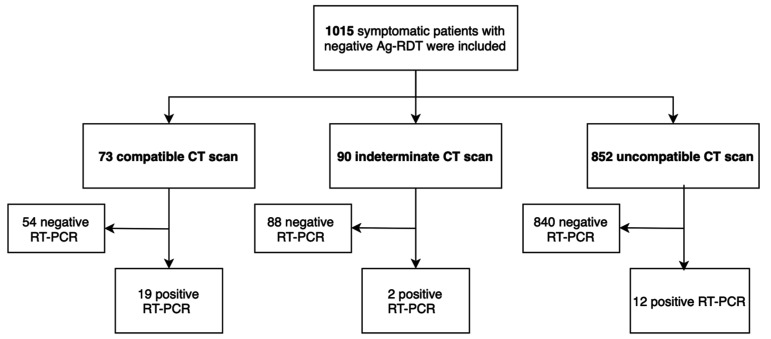
Results of combined strategy compared with RT-PCR.

The negative predictive value of Ag-RDT alone was 96.7% compared to RT-PCR (regardless of Ct values), with 3.3% (33/1015) false negatives (95% CI 2.3–4.5). The negative predictive value of combining Ag-RDT/CT was 98.5% compared with RT-PCR (Ct < 40), with 1.5% (14/942) false negatives (95% CI 0.9–2.4) (Figure 3). Interestingly, when only RT-PCR results with low viral load were considered (Ct values > 30), the negative predictive value of combining Ag-RDT/CT increased to 100%, with 0% (0/1015) false negatives (95% CI 0.0–0.2).

Overall, the addition of the CT strategy was more likely to identify patients with COVID-19 in the ED and helped detect 58% (19/33) of false negatives.

Predictive negative value: 98.5% (928/942). Combined strategy positive: Chest CT compatible and negative Ag-RDT. Combined strategy negative: Chest CT indeterminate or incompatible and negative Ag-RDT.

## 4. Discussion

Delays in receiving RT-PCR results can be long and have a direct impact on hospital organization in the absence of an efficient and rapid triage strategy in the ED. However, the delay for Ag-RDT in the ED was 15–20 min, which allowed a decrease in time to clinical decision. Our study revealed that combining Ag-RDT is successful in ruling out COVID-19 for 98.6% of patients with COVID-19-like symptoms but negative Ag-RDTs. It has to be noted that all patients with viral loads ≤ 30 Ct were identified in our cohort with high precision.

Our results revealed better negative predictive value than a previous study with Ag-RDT [14,15,16]. The duration of symptoms was shorter in our study for patients with a compatible CT than those with incompatible or indeterminate CTs, but this data was not well informed in our retrospective study. Most studies revealed that Ag-RDT performed well in the first 7 days after symptoms onset, with declining performance after more days [17,18]. Indeed, CT is of interest for identifying patients with COVID-19 in the ED. The lower sensitivity of the scanner in the first 3–4 days, the period during which viral load is highest and therefore when the Ag test has its best sensitivity, makes the combination of these 2 techniques relevant. A prospective survey conducted with 7500 patients from 2 March 2020 to 24 April 2020, the period that corresponded with the peak of the French national COVID-19 epidemic, revealed a chest CT sensitivity of 90% (95% CI 88–91; 2320/2564) [19]. In our study, more than half of the false negative Ag-RDTs with positive RT-PCRs were identified by CT. Among other patients in which CT was not discriminant (2 indeterminate and 12 incompatible CTs), no patients had a positive RT-PCR with Ct ≤ 30. The Ct  ≤ 30 threshold was recommended by the German Robert Koch Institute as a viral infectivity threshold for identifying SARS-CoV-2-contagious individuals [20,21]. We therefore considered this Ct value as a cutoff for stratifying patients with low (Ct > 30) or high (Ct ≤ 30) viral load. The sensitivity of Ag-RDT decreased as viral load decreased with Ct < 30 (from 93.5% to 33%) [17]. Thus, all patients with high viral loads were identified by the combined strategy in this cohort.

According to the recommendations of the WHO, Ag-RDT offers the possibility of rapid, inexpensive, and early detection of COVID-19 [22]. Indeed, the WHO Emergency Use Listing detailed priority categorization for in vitro diagnostics and recommended two Ag-RDTs, including the Panbio™ COVID-19 Ag Rapid Test Device used in our study [23,24]. Even if Ag-RDT is useful in the ED for early identification of COVID-19, the number of false negatives requires confirmation by RT-PCR and isolation of patients [16]. In our study, 549 patients with indeterminate or incompatible CT findings were hospitalized in the ED short-stay unit awaiting their RT-PCR results, which were positive for only seven of the patients, all harboring low viral load. This study highlights a key potential strategy that could reduce ED stays and improve conditions of overcrowding in these frontline patient triage centers. Ag-RDT combined with CT facilitates accurate and rapid COVID-19 detection in EDs to control hospital transmission, an effective strategy that immediately transfers patients to the most appropriate department. This strategy correctly identifies patients with clinical symptoms and allows their transfer to a single hospital room until their RT-PCR results are available. Mixed-care units could therefore be proposed as an efficient option following the first level of triage to ED.

The main limitation of the current study is that although the prevalence was 10.06%, it was not as high as in the peak of the pandemic. However, given the high negative predictive value, we can extrapolate that the combined strategy is effective in ruling out the diagnosis of COVID-19 in the ED, even in conditions of higher prevalence. Another limitation could concern the period of this study, before occurrence of the most infective Delta variant. However, a recent publication of the Chinese Center for Disease Control and Prevention showed that the viral load of the first positive tests of Delta infections was about 1000 times higher than that of the wild-type virus during the initial pandemic in 2020, suggesting a potentially faster viral replication rate, which would increase the sensitivity of Ag-RDT [25]. Furthermore, Ag-RDT accurately detects the new viral variants, notably when devices are operated by trained medical staff [26]. Hence, the strategy of combining Ag-RDT and CT probably would be more relevant to rule out COVID-19 in patients with COVID-like symptoms in the ED.

## 5. Conclusions

A strategy combining Ag-RDT and chest CT is effective in ruling out COVID-19 infection and can reduce ED stays, with direct admission to medical wards instead of short-stay medical units while awaiting RT-PCR results. However, the results have yet to be interpreted based on patients’ clinical signs.

## Figures and Tables

**Table 1:** Characteristics of the study population.

Characteristics		N (%), M (SD) N = 1015
Age		70.4 (18.2)
Gender	Men Women	557 (54.9) 458 (45.1)
PCR positive before ED stay		25 (2.5)
Contact with COVID before ED stay		6 (0.6)
Chest CT	Compatible	73 (7.2)
	Indeterminate	90 (8.9)
	Incompatible	852 (83.9)
PCR positive in ED		33 (3.2)
Hospitalization in medical ward		600 (59.1)

CT: Computed Tomography; ED: Emergency Department.

## Data Availability

Restrictions apply to the availability of these data. Data are available from the authors with the permission of Hopitaux Universitaires de Strasbourg.

## Abbreviations

COVID-19	coronavirus disease 2019
ED	emergency department
Ag-RDT	antigen-detecting rapid diagnostic tests
CT	computed tomography
